# The Relationship Between Specific Age-Related Chronic Conditions of Comorbidity and Depression Scores Among Men in an Elderly Population: A Cross-Sectional Study

**DOI:** 10.7759/cureus.28000

**Published:** 2022-08-14

**Authors:** Yousef Waly, Muhammed Hussain, Mohamed Shelig, Ahmed Al-Hindawi, Ali Al-Sabti, Yahya Al Farsi

**Affiliations:** 1 Medicine and Surgery, Royal College of Surgeons in Ireland - Bahrain, Manama, BHR; 2 Medicine and Surgery, University College Dublin, Dublin, IRL; 3 Medicine, Sultan Qaboos University, Muscat, OMN

**Keywords:** diabetes, arthritis, chronic lung disease, congestive heart failure, tilda, cancer, elderly population, chronic diseases of comorbidity, depression

## Abstract

Objective

There had been an observed increase in the prevalence of depression as well as many chronic conditions of comorbidity among the elderly population of Ireland above the age of 50. The relationship between different prominent conditions of comorbidity and depression scores amongst older adult men in Ireland was sought to be examined and explored.

Methods

A cross-sectional analysis of data from wave 1 of The Irish Longitudinal Study on Aging (TILDA) had been used for statistical analysis, which served to be the representative cohort study sample of elderly adults living in Ireland aged 50 and older. Summary statistics (cross-tabulation, t-test, analysis of variance/ANOVA and odds ratio) were used to explore the relationship between depression scores and different conditions of comorbidity.

Results

Results were drawn from the three different tests conducted; cross-tabulation, t-test, and analysis of variance/ANOVA. Cross-tabulation served to provide the total population of men who suffered from depression (CES-D score ≥ 16), which totaled 123 (1.4%) of the entire 8,504 available candidates. Of the participants that met the criteria for having a significant risk of clinical depression along with an accompanying chronic illness odds ratio (OR) had been calculated. All but one of the conditions yielded a significant increase in OR between having a chronic condition and depression; with the exception of chronic lung disease. Congestive heart failure demonstrated the highest OR of 4.40 (CI 95% 1.77-10.95), followed by arthritis, diabetes and cancer. Subsequent t-tests used to construct an ANOVA then illustrated the mean CES-D score for males suffering from one of the five concomitant illnesses selected (congestive heart failure, chronic lung disease, arthritis, cancer, and diabetes) as well as those free of the selected diseases of the study, with a total count of 2,117. All results had been deemed to be significant with p*-*values < 0.05, with men suffering from congestive heart failure having the highest mean score of 7.28 (n=39). Those who do not suffer from any of the five conditions reported the lowest scores and also accounted for the largest population group with 3.88 and 1,387, respectively.

Conclusions

Consistent and significant findings of elderly men suffering from a chronic condition of comorbidity demonstrated having elevated OR and CES-D scores in comparison to those who are disease-free. The findings of this study can be used to evaluate alternative preventative management of chronic diseases of comorbidity in order to improve the depression scores of patients.

## Introduction

Depression is a public health problem that has rapidly increased in prevalence, affecting approximately 280 million people worldwide according to the World Health Organization (WHO) [1]. In addition, previous studies have projected depression to become the leading cause of the burden of disease by the year 2030 [2]. Furthermore, there has been a rapid rate of depression prevalence and burden amongst low and middle-income countries (LMICs) as a result of the quickly changing transitions in social culture as well as the increased risk of “western” lifestyle risk factor exposure [3]. If depression is not adequately treated, chronic depression would most likely lead to increased disability, with a comparable burden to physical disability [4].

According to the National Council of Aging (NCOA) [5], there has been an increase in the prevalence of conditions of comorbidity amongst the elderly population, such as congestive heart disease, lung disease, arthritis, cancer, and diabetes. In addition, such conditions have mostly been found to be more prevalent among men in an elderly population. Depression has been suggested to be a possible complication of chronic conditions of comorbidity [6]. However, the pathophysiological as well as behavioral mechanisms behind this are yet to be precisely identified but are proposed to be due to the activation of the stress axis, hypothalamic-pituitary-adrenal (HPA) axis dysregulation, immune-mediated inflammation, and the effects of serotonin [7].

Recent studies have indicated that men are considerably less likely to report and even be diagnosed with depression due to social and cultural stigmas, leading to the subconscious tendency of practitioners to overlook male distress. Such association between chronic conditions of comorbidity prominent in the elderly population of men and depression must be evaluated and explored [8]. Hence, the question lies: What is the relationship between specific age-related chronic conditions and mean depression scores among men in an elderly population?

Therefore, this study aims to examine the relationship between the different aforementioned chronic conditions of comorbidity and depression scores in a more extensive sample study among male patients. It is hypothesized that through this larger sample size, the influence of different chronic conditions of comorbidity on the severity of depression, quantified using reported Center for Epidemiologic Studies Depression Scale (CES-D) scores, could be identified.

## Materials and methods

Study and sample 

A compiled data record from wave 1 of The Irish Longitudinal Study on Aging (TILDA) study [9] includes a representative clustered sample of 8,504 individuals aged 50 years and above in Ireland. This dataset is granted access to and permitted for statistical analysis and publication use via Trinity College Dublin. TILDA was conducted between the months of October 2009 and February 2011, with data collection compromising both face-to-face interviews as well as self-completion questionnaires. Table 1 illustrates the demographic data of the overall study participants demonstrating different aspects such as age, education, employment status, etc.

**Table 1:** Demographic data of wave 1 TILDA participants TILDA - The Irish Longitudinal Study on Aging

Variable	Categories	n (%) or mean (SD)
Age	Mean age in years	62.97 (± 9.4)
	25-49	329 (3.9%)
	50-54	1,662 (19.1%)
	55-59	1,649 (19.4%)
	60-64	1,393 (16.4%)
	65-69	1,196 (14.1%)
	70-74	963 (11.1%)
	75-79	714 (8.4%)
	80-84	626 (7.4%)
Sex	Male	3,780 (44.4%)
	Female	4,724 (55.6%)
Education	Some primary (not complete)	280 (3.3%)
	Primary or equivalent	2232 (26.2%)
	Intermediate/junior/group certificate or equivalent	1971 (23.1%)
	Leaving certificate or equivalent	1460 (17.2%)
	Diploma/certificate	1335 (15.7%)
	Primary degree	730 (8.6%)
	Postgraduate/higher degree	483 (5.7%)
Employment	Retired	3048 (35.8 %)
	Employed	2218 (26.1%)
	Self-employed (including farming)	923 (10.9%)
	Unemployed	413 (4.9%)
	Looking after home or family	1346 (15.8%)
	Other	554 (6.4%)
Self-rated physical health	Excellent	1360 (16.0%)
	Very good	2448 (28.8%)
	Good	2758 (32.4%)
	Fair	1517 (17.8%)
	Poor	420 (4.9%)
Self-rated mental/emotional health	Excellent	2255 (26.5%)
	Very good	2927 (34.4%)
	Good	2475 (29.1%)
	Fair	725 (8.5%)
	Poor	120 (1.4%)

Professionally trained interviewers were involved in collecting data on health, social, and economic factors from all study participants. In this study, male participants who either reported only one of the selected chronic conditions (congestive heart disease, lung disease, arthritis, cancer, and diabetes) or no reported conditions had been analyzed. Through this exclusion criteria, a total of 730 male participants were identified to have reported having only one of the aforementioned conditions, whereas 1,387 male participants reported no disease. As a result, a total of 2,117 male participants (ages ranging between 50 and 80+) have been included for analysis against the reported CES-D depression scores.

Measures

Depression Score

During wave 1 of the TILDA study, symptoms of depression were assessed and analyzed using the CES-D tool published by Radloff in 1977 [10]. The self-reported CES-D tool provided high coefficients of reliability [11], which ranged between 0.85 and 0.91 when used among the elderly demographic of adults in Ireland. The instrument is comprised of a series of questions on depression symptomatology. Symptoms were scored on a scale of 0 to 3, ultimately tallying up to a score out of 60. Scores of ≥16 indicate patients who are at risk of having clinical depression. Assessment of this cut-off score in the elderly demographic reported a sensitivity of 100% and a specificity of 87.6% [12]. In addition, the reported negative predictive values were deemed to be as high as 100% while the reported positive predictive values were found to be as low as 13.5% [12]. In other words, individuals scoring under the 16-score cut-off are unlikely to be diagnosed with depression.

Chronic Conditions of Comorbidity

Surveyed participants were asked if they had received the diagnosis of congestive heart disease, lung disease, arthritis, cancer, diabetes, or none of the listed diseases from a doctor. These were then set to serve as the independent variables of this study. The specific selection of these variables had been based on the common prevalent chronic conditions seen in the elderly demographic reported by literature, as well as the availability of variables found within wave 1 of the TILDA dataset.

Analysis

Summary statistics for all variables of this study had been conducted. Male patients in the aforementioned independent variable groups (congestive heart disease, lung disease, arthritis, cancer, diabetes, or none) were selected based on the criteria of being diagnosed with only one of the previously mentioned conditions of this study or having no diseases diagnosed which was indicated by “none” in the dataset.

Firstly, only male patients with an indication of being at risk of clinical depression (CES-D score ≥ 16) between the different selected disease groups had been assessed via the cross-tabulation test. Furthermore, ​​mean depression scores without having a cut-off score are implemented for each of the selected chronic conditions of comorbidity and were then compared to “none” via t-tests. After several t-tests had been conducted for each of the specific chronic conditions of comorbidity, the t-tests were compiled into an ANOVA test in order to further compare mean depression scores between all independent variables against each other simultaneously; allowing for more potential connections to be explored and drawn out in a more holistic manner. Lastly, odds ratios (OR) had been calculated between the different chronic conditions and disease-free patients with CES-D scores ≥ 16, in order to observe the associated odds of developing symptomatology of clinical depression. All statistical tests and analyses had been conducted via the SPSS software for Macintosh version 28 (IBM Corp., Armonk, NY).

## Results

Within the study, 123 participants met the criteria of both being male and at risk for clinical depression (CES-D ≥16). This accounted for approximately 1.40% of the total participants (n=8,504) or 3.25% of the total male participants (n=3,780). When comparing the CES-D cut-off score against chronic conditions such as chronic lung disease, arthritis, cancer, diabetes, and congestive heart failure, it was shown that the largest portion of participants did not present with the chosen chronic conditions; these people accounted for 44.7% (n=55) of the total 123 that were being analyzed. Males who suffered from concomitant arthritis accounted for the next largest portion at 26.0% (n=32), followed by diabetes at 13.0% (n=13), as illustrated in Table 2.

**Table 2:** Population of males with clinically diagnosed depression (CES-D score ≥16) with or without any chronic conditions

	Number of male patients with CES-D scores ≥16 (Depression)	% of total
Arthritis	32	26.0%
Congestive Heart Failure	6	4.9%
Diabetes	16	13.0%
Cancer	10	8.1%
Chronic lung Disease	4	3.3%
None of these	55	44.7%
Total	123	100%

Following the conduction of the cross-tabulation to determine the population suffering from clinically diagnosable depression via the CES-D score metric, OR had been calculated to evaluate the potential influence of different exposures/chronic conditions on the risk of developing clinical depression (CES-D score ≥ 16). High OR of each of the five selected conditions had been demonstrated, however, all but one condition were seen to be statistically significant with the exception being chronic lung disease which had a p-value >0.05 (0.630) (Table 3). While all chronic conditions demonstrated high OR, congestive heart failure demonstrated a far higher association than any of the other conditions with an OR of 4.40 (CI 95% 1.77-10.95), which is significantly higher than the second-highest OR of 2.66 (CI 95% 1.69-4.19) by arthritis. Each of the other significant findings (p-value > 0.05) still showed a strong association with all having OR of over 2, meaning people suffering from these conditions were more than two times more likely to have an overlapping diagnosis of depression.

**Table 3:** Odds ratio values of males with chronic conditions calculated against disease-free patients with CES-D Scores ≥ 16

	Odds Ratio (OR)	95% Confidence Interval	p value
Arthritis	2.66	1.69 - 4.19	<0.05
Congestive Heart Failure	4.40	1.77 - 10.95	<0.05
Diabetes	2.65	1.48 - 4.75	<0.05
Cancer	2.07	1.03 - 4.17	<0.05
Chronic lung Disease	1.29	0.456 - 3.66	0.630

Several t-tests had then been conducted for each of the selected chronic conditions against each of the individual’s reported CES-D scores to illustrate the individual correlation. The conducted t-tests had then been compiled into an analysis of variance (ANOVA), in order to view the variables all at once in a comparative measure. All results of the ANOVA were found to be significant with the p-value being found to be at <0.001, indicating statistical significance amongst the data. As illustrated in Table 4, the mean CES-D scores of each group can be identified and compared to the control of males who are not suffering from any of the five selected concomitant illnesses. The total males that met the study metrics criteria totaled 2,117 with an individual average CES-D score of 4.70. The largest proportion of the study sample fell into the category of “none of the above,” accounting for 65.5% (n=1,387) of the total study sample.

**Table 4:** Association between mean CES-D scores and presence of concomitant chronic conditions

	n (% total)	Mean CES-D scores	95% CI	Overall ANOVA p-value
Arthritis	323 (15.26%)	6.44	5.60 - 7.28	<0.001
Congestive Heart Failure	39 (1.84%)	7.28	5.06 - 9.50	
Diabetes	162 (7.65%)	6.26	5.17 - 7.35	
Cancer	127 (6.00%)	5.61	4.42 - 6.79	
Chronic lung Disease	79 (3.73%)	6.08	4.56 - 7.59	
None of these	1387 (65.52%)	3.88	3.62 - 4.15	
Total	2117	4.70	4.45 - 4.96	

The relationship between males suffering from congestive heart failure and their respective CES-D scores was the highest of any of the other chronic conditions, at an average of 7.28 (SD=6.85 CI 95% 5.06-9.50). While males with concomitant congestive heart failure presented with the highest average of CES-D scores, the values also did have the largest standard error of the groups (SE=1.10), the only condition found to be >1. This could possibly be correlated to the fact that the dataset offered the smallest group sample size (n=39) for congestive heart failure and therefore the largest variation of presented values. Expectedly, the group of people that did not have any of the other chronic conditions (n=1387) reported the lowest mean CES-D score at 3.88 (SD= 5.07 Cl 95% 3.62-4.15). The remainder of the groups showed very little distribution and were within 1 CES-D score of each other, ranging from 5.61-6.44, with the lower end belonging to males with cancer (n=127) and the higher end males with arthritis (n=323). Males suffering from a chronic condition consistently reported higher CES-D scores than those who were disease-free, as indicated in Table 2. These elevated CES-D scores indicate the correlation of depressive effects or feelings on those experiencing chronic conditions.

## Discussion

Main findings

Within the findings of this study, a relationship had been established between the different chronic conditions and the risk of depression as further backed by the statistically significant results (p-values <0.001). Congestive heart failure, a type of cardiovascular disease, presented with the highest mean CES-D score (7.28) as well as the highest OR (4.40). This was seen to be consistent with previous studies, which have portrayed cardiovascular disease to be associated with the most severely elevated levels of depression when compared to other chronic conditions [7]. After congestive heart failure, in an order of highest to lowest CES-D scores respectively, the results were arthritis, diabetes, chronic lung disease, and cancer.

In terms of the OR, the order of likelihood of having depression (CES-D score ≥ 16) correlates mostly with the mean CES-D scores between the different chronic conditions. The exception, in this case, is between patients with chronic lung disease and cancer, in which patients with cancer had a lower mean CES-D score, but a higher OR indicating it is more likely for cancer patients to have symptomatology of clinical depression compared to patients with chronic lung disease. However, the OR value for patients with chronic lung disease was insignificant (p > 0.005).

The ranking of arthritis as having the most severe CES-D score (after CVD) may be attributed to the established fact that sufferers of the disease may not be able to engage in activities or hobbies that they once enjoyed, especially those that are physically strenuous. With males having higher engagement levels in leisure-time physical activity, it is expected that the arthritic effects leading to a substantial decrease in physical activity among men will likely lead to higher levels of depression [13].

Chronic conditions other than congestive heart failure tend to display varying results in terms of depression scores in compared studies. The ranking of depression severity between diabetes, chronic lung disease, and cancer was seen to vary in different studies that compared various chronic conditions and depression. In terms of the dataset used in this study, the order of diseases may be attributed to factors such as sample size. The number of respondents per chronic condition varied significantly between the different categories, ranging from 39 sampled with congestive heart failure, to 323 men with arthritis.

Lastly, amongst all male patients assessed, only 123 had been assessed to likely have true clinical depression (CES-D ≥16). Of the 123 patients, arthritis was seen to be most prevalent (26%) among all other explored chronic conditions. This may allude to the previously suggested decrease in physical activity leading to the increased risk of being diagnosed with clinical depression. In retrospect, congestive heart failure (4.9%) and chronic lung disease (3.3%) were seen to be the least prevalent. While the CES-D scores of the aforementioned conditions ranked higher, an exploration into why the prevalence of clinical depression is lower is warranted.

Strengths and limitations

This study comes with a number of both strengths and limitations. Beginning with strengths, many of the patients that have been studied are dealing with multiple chronic conditions simultaneously. Through the use of the TILDA data set, patients that belonged to multiple categories of chronic conditions were controlled in order to provide more precise data as well as reduce the influence of confounding factors. This leads to more apparent connections to be drawn between the severity of depression and the type of chronic conditions at hand. However, despite controlling for these factors, other confounding factors have not been factored in, which may potentially affect levels of depression other than chronic conditions. Examples include socioeconomic factors such as income and education levels, as well as social support. In fact, it has been found that those with lower socioeconomic status (SES) are likely to have higher rates of depression, which may have an effect on CES-D scores [14]. In terms of other weaknesses, it is not possible to establish a causal relationship between the findings since this was a cross-sectional study. Perhaps a more direct means, such as a cohort study, may be used to explore the presence of causal relationships. In addition, some categories of chronic conditions contained a relatively small number in the sample sizes when compared to the rest. This is unfortunately due to the limited number of patients reporting only one condition available within wave 1 of the TILDA dataset that was able to be included in this study. Hence, it would be appropriate to evaluate a larger sample size to reduce standard error in future studies.

Implications

It is evident that depression and other mental health disorders disproportionately affect victims of certain illnesses over others. Based on the data, it has been illustrated that patients with congestive heart failure have depression scores higher than any other illness, as well as almost double the severity of depression in comparison to patients without chronic conditions. It is also noted that cancer patients had the lowest depression scores on average compared to the remaining groups of diseases. Potential reasoning for this statistic could be attributed to the stronger social support systems that cancer patients tend to have. Research findings have demonstrated that a strong relationship exists between the level of social support and the progression of cancer, emphasizing the importance of having a strong social network during cancer treatment [15]. Similar links have also been established for other chronic illnesses. In fact, depression is a strong predictor of disease progression and prognosis in those suffering from chronic conditions [16]. An increase in mortality rates, as well as a decrease in quality of life (QOL), has been commonly observed among depressed patients diagnosed with chronic conditions such as heart disease, chronic obstructive pulmonary disease (COPD), and diabetes [16]. Accordingly, promoting the importance of developing strong social support systems for those with chronic illnesses, to the same degree as those with cancer, may seek benefit in improving both the overall outcome of disease as well as psychological wellbeing.

Furthermore, when it comes to depression, men seek help from a professional at half the rate of women and therefore have a quite significant rate of undiagnosed depression compared to their female counterparts [16,17]. Therefore, it is immensely crucial to not only implement concurrent management of depression alongside treatment of chronic disease but also to put into practice the use of screening tools for depression within the clinical setting. Use of depression screening tools followed by appropriate therapeutic management results in the improvement of both the perceived quality of life and depression in patients with chronic illnesses, which has been indicated in a study conducted by Jensen et al. [18]. Additionally, earlier diagnosis and treatment of depression have been shown to provide favorable outcomes in terms of the rate of relapse and remission [19]. Hence, within a clinical environment, the application of depression screening tools has the potential to increase the rate of depression diagnosis and improve the prognosis of both depression and concomitant chronic disease, especially when used early on within disease progression.

## Conclusions

In conclusion, the findings of this study align with the intended objective. A consistent relationship was seen between men suffering from chronic health conditions such as congestive heart disease, lung disease, arthritis, cancer, and diabetes against depression. The effect of suffering from a chronic condition may alter one’s regular way of life and subsequently affect their mental state of mind as visualized by the rise in CES-D scores. This was also seen to be noticeable by the population that did not suffer from any of the other diseases having the lowest mean CES-D score by a significant margin. The study illustrated the effects of specific chronic conditions on depression and indicated that concomitant congestive heart failure patients presented with the highest average CES-D scores. In addition, OR had been calculated between each of the chronic conditions of comorbidity against disease-free patients with CES-D scores ≥ 16. All ratios portrayed significantly elevated odds of developing symptomatology of clinical depression given the patient had one of the selected chronic conditions, with the exception of chronic lung disease demonstrating statistical insignificance. Overall, this study provides legitimate indications of the relationship between depression arising in chronic condition sufferers. While a study like this has not exactly been conducted previously, this will set to provide the means for further exploration and investigation in the future through cohort studies to truly dissect causal relationships.
